# A screw view model of navigation guided minimal invasive percutaneous pelvic screws insertion for lateral compression pelvic ring injuries: A case report

**DOI:** 10.1097/MD.0000000000021755

**Published:** 2020-10-02

**Authors:** Bao-Ming Yuan, Ge Huang, Shuang Zheng, Tong Yu, Jian-Wu Zhao

**Affiliations:** a Department of Orthopaedics; b Department of Radiology, The Second Hospital of Jilin University, Changchun, Jilin Province, China.

**Keywords:** anterior inferior iliac spine screw, pelvic ring injuries, percutaneous, screw view model of navigation

## Abstract

**Rationale:**

The objective of the present study was to evaluate the accuracy, effectiveness, and safety of screw view model of navigation (SVMN) guided minimal invasive percutaneous pelvic screws (PPSs) insertion for lateral compression pelvic ring injuries (PRI).

**Patient concerns:**

A female patient experienced a high falling injury, and presented with pain, swelling, deformity, and movement limitation of the left hip for 3 hours.

**Diagnoses:**

She was diagnosed with pelvic fractures, left iliac fracture, left pubic branch fracture, left ischial branch fracture, and lumbar transverse process fracture.

**Interventions:**

We used a SVMN technique to guide PPSs insertion, including a percutaneous anterior inferior iliac spine screw, a percutaneous iliac screw (PIS), and a percutaneous sacroiliac screw (PSIS).

**Outcomes:**

In total, 3 PPSs were inserted and all were presented with excellent position postoperatively. The designing time of all screws was 11.7 minutes, the time of all guide needles insertion was 18.1 minutes, the time of all screws insertion was 32.8 minutes, blood loss was 21 mL, and the time of radiation exposure lasted 7.2 minutes. Moreover, surgical complications, including neurovascular compromise, wound infection, fracture nonunion, and screw loosening, were not observed during the 12 months follow up visit.

**Lessons:**

SVMN technique guided PPSs insertion is an effective and safety approach for the treatment of PRI in selected patients. Besides, it is necessary for surgeons to master the rationale of computer navigation, to familiar with the anatomy of pelvis and to select suitable patients.

## 1 Introduction

Pelvic ring injuries (PRI) commonly result from high energy trauma, such as falling injury and motor vehicle accident injury. According to previous studies, 5% to 20% of the patients experienced severe hematoma, and 10% to 31.1% of the patients suffered death.[^1^,^2^,^3^,^4^] Among the PRI, lateral compression fractures are the most frequently injuries, which are occasionally reducing closed reduction, and usually vertically stable.[^5^,^6^] In the past, the management of PRI with bed rest and delayed weight bearing has been advocated.[^7^,^8^,^9^] The long-term target of treating pelvic ring fractures is to correct deformity, prevent deformity and instability postoperatively, and restore pain-free function,^[10]^ and early dynamic exercises. In recent years, the management strategies for PRI mainly including reduction and internal fixation through open or closed approach,^[11]^ anterior subcutaneous internal fixation,^[12]^ and anterior pelvic external fixation.^[13]^ Nevertheless, these techniques have many surgical complications, such as nerve compromise, wound infection and deep infection, fracture nonunion, confusional states postoperatively, anemia, urinary tract infection, and pneumonia.[^3^,^14^]

Currently, percutaneous pelvic screws (PPSs) fixation is recommended due to the character of stable fixation, minimal incision, rapid recovery, less blood loss, and short hospital stay. However, PPSs insertion regarded technique demanding because the complexity of the pelvis anatomy. Moreover, the surgeons and patients with prolonged radiation exposure.[^15^,^16^] Many strategies have developed to facilitate PPSs insertion for PRI, such as a robot-assisted navigation system, a computed tomography (CT)-based navigation and a fluoroscopic-based navigation.[^1^,^17^,^18^,^19^,^20^] Nevertheless, there are still occasional surgical complications, such as neurovascular damage and wound infections. Consequently, the purpose of the present study is to evaluate the accuracy, effectiveness, and safety of screw view model of navigation (SVMN) guided PPSs insertion for the treatment of lateral compression PRI.

## 2 Ethical approval

This report was approved by the ethics committee of the Second Hospital of Jilin University, Changchun, China. The patient a provided written informed consent for this report, and we maintained his anonymity.

## 3 Case report

### 3.1 Patient characteristics

A 35-year-old female patient experienced a high falling accident (Table 1), and presented with swelling and pain of left hip. Physical examinations showed that pelvis compression and separation test were positive.

**Table 1:**
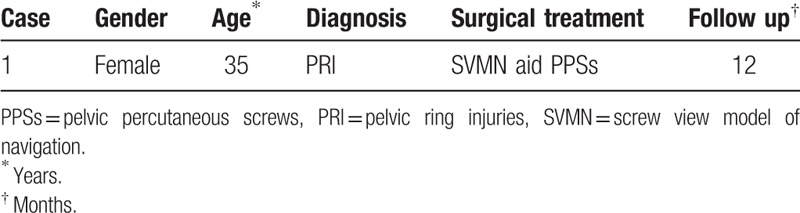
Basic characteristics of the patient.

**Figure 1:**
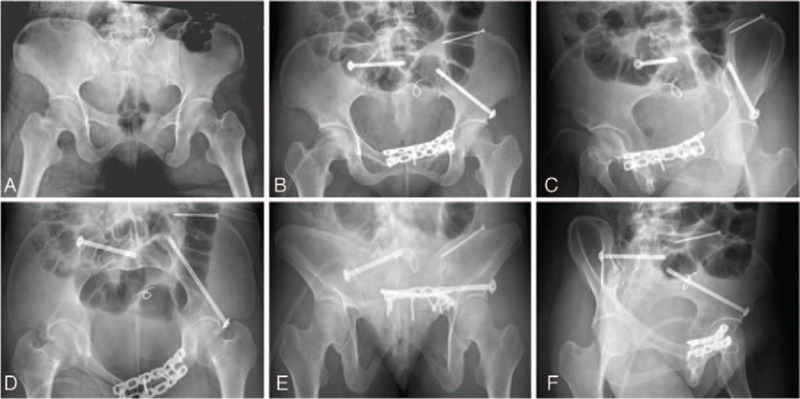
(A) Preoperative radiograph showed that there was bone discontinuity and displacement in the left superior and inferior pubic ramus, and a gap was showed though the ilium. (B–F) Postoperative radiographs demonstrated that the positions of PPSs were excellent. PPSs = percutaneous pelvic screws.

**Figure 2:**
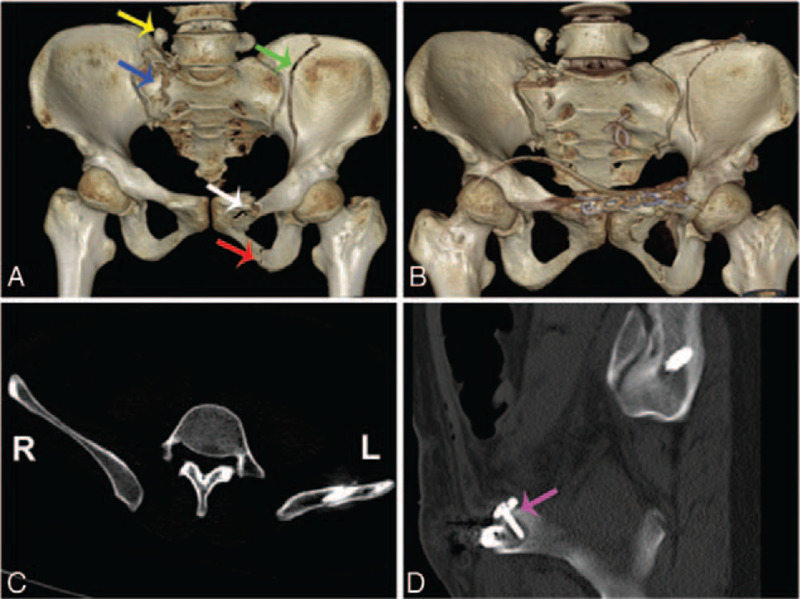
(A) Preoperative 3D CT showed that there was bone discontinuity and displacement in the left superior (the white arrow) and inferior (the red arrow) pubic ramus, and a gap was showed though the ilium (the green arrow). The sacrum (the blue arrow) was not in regular shape and the right transverse process of the L5 vertebral (the yellow arrow) was also found to be irregular. (B) Postoperative 3D CT scanning. (C) Postoperative CT image showed that anterior inferior iliac spine screw was through the fracture line and entirely in cancellous bone. (D) Postoperative CT image revealed that the iliac screw was in good position (the purple arrow). 3D = three dimensional, CT = computed tomography.

In accordance with the preoperative radiograph (Fig. 1A) and three dimensional (3D) CT scanning images (Fig. 2), there was bone discontinuity and displacement in the left superior and inferior pubic ramus, and a gap was showed though the ilium. The sacrum was not in regular shape, and the right transverse process of L5 was also found to be irregular. The patient was diagnosed with PRI, including left pubic ramus fracture, iliac fracture, sacroiliac fracture, and transverse process fracture of the L5.

### 3.2 Surgical technique

The patient was placed in the supine position once the anesthesia took effect.

#### 3.2.1 Image acquisition

A patient tracker (Stryker Leibinger GmbH & Co., Freiburg, Germany) was fixed on the right iliac crest. After the patient tracker, C-arm tracker, and instruments tracker were all opened, a 190° scan was performed at the center of the pelvis fractures and then image date of the fracture site were achieved.

#### 3.2.2 Surgical planning

**Figure 3:**
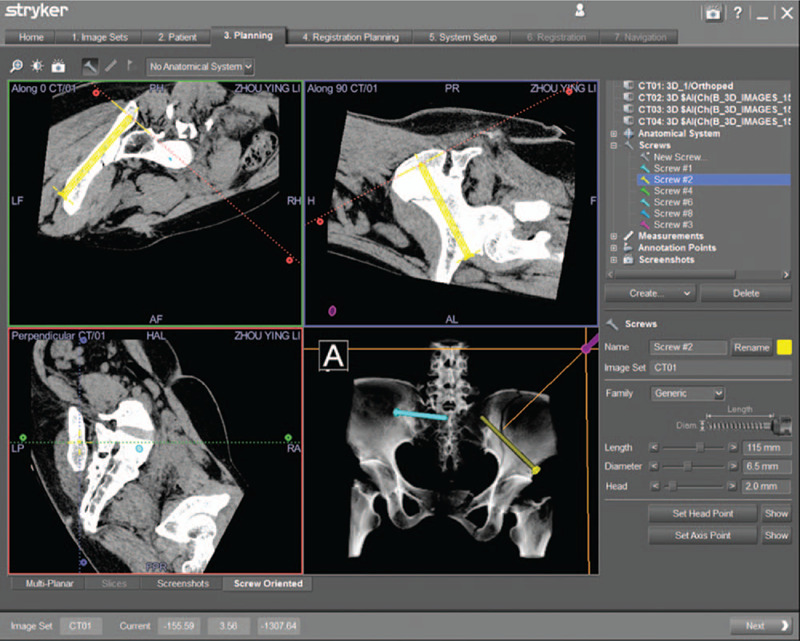
A percutaneous anterior inferior iliac spine screw was designed to fix the iliac fracture along the anterior inferior iliac spine to the posterior superior iliac spine preoperatively. The screw details including the length, the diameter, and the direction were also show in navigation workstation.

The work station of navigation system provided multi-planar images of the pelvic fractures, which contribute to design the PPSs, including the length of screw, the diameter of screw, and the optimal direction for screw (Fig. 3). In principle, the inserted 3 PPSs were not perforating bone cortex and through fracture line. A percutaneous anterior inferior iliac spine screw (PAIISS) was designed to fix the iliac fracture along the anterior inferior iliac spine to the posterior superior iliac spine, and a PIS screw was planned and passed through the fracture line from left iliac wing. Besides, a percutaneous sacroiliac screw (PSIS) screw was also designed and inserted, details reference to Yu et al.^[21]^

#### 3.2.3 Screw implantation

**Figure 4:**
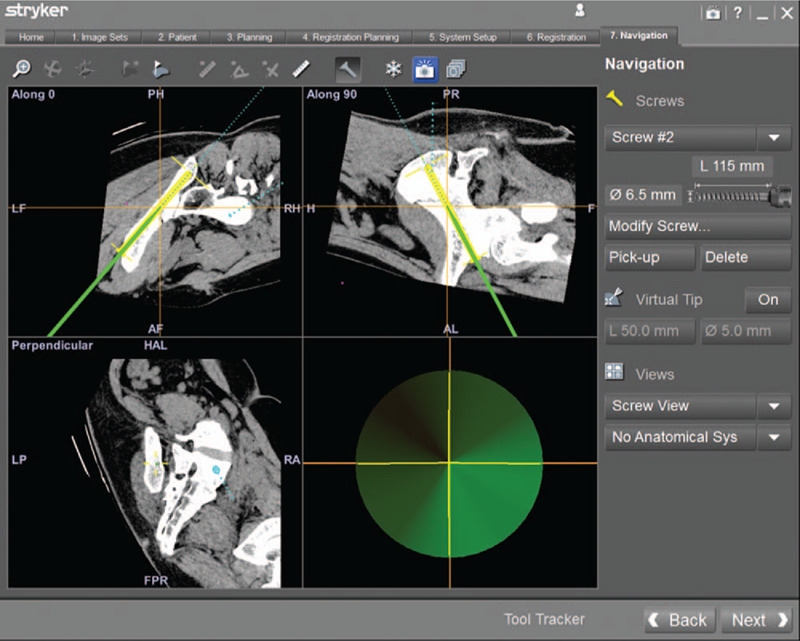
The image of the right lower corner shows green color, which indicates the optimal moment to insert a guide needle from the anterior inferior iliac spine.

**Figure 5:**
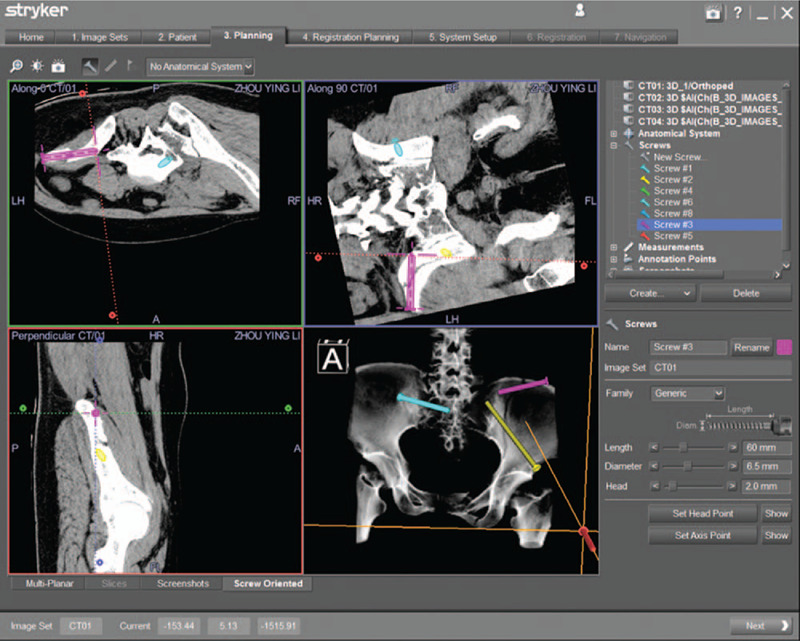
A percutaneous iliac screw was planned preoperatively. The screw details including the length, the diameter, and the direction were also show in navigation workstation.

**Figure 6:**
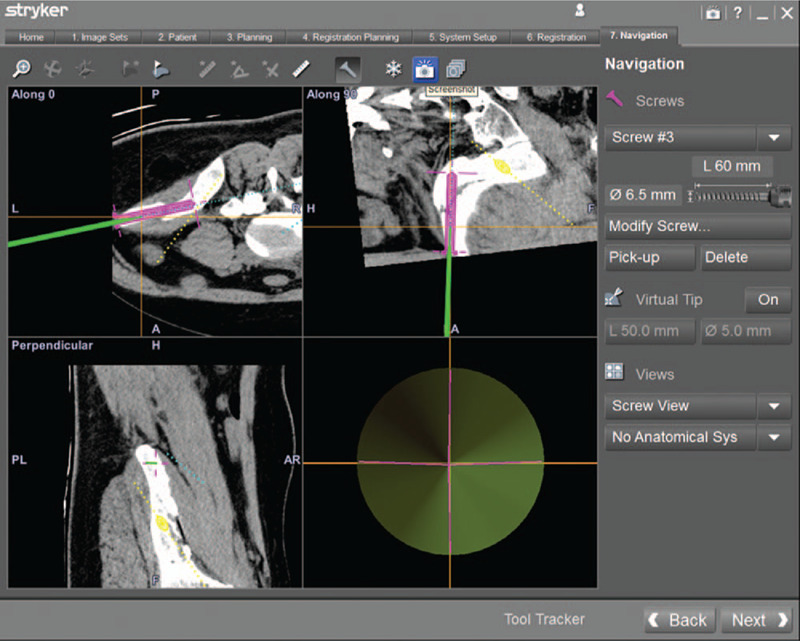
A guide needle of iliac screw was inserted.

The screw view model was selected in the workstation of navigation system (Fig. 3). Intraoperatively, the position of guide needle cannular was continuously updated by the infrared camera, and multiplanar images displayed on navigation monitor simultaneously, which allowing real-time feedback of the direction of guide needle cannular and the planned screws preoperative. The trajectory of the guide needle cannular was adjusted until the position of the surgical instrument was completely in accordance with the designing direction of anterior inferior iliac spine screw and iliac screw. It is the optimal time to insert the guide needle once the image in the lower right corner of the navigation monitor is green (Fig. 4). Then the anterior inferior iliac spine screw and iliac screw were inserted (Figs. 5 and 6).

### 3.3 Outcomes and follow-up

We evaluated the position of the inserted screw according to Gras et al,^[19]^ and the location of placed screws were classified as 3 grades: Grade I (the screw was completely within in the cortex bone), Grade II (the screw cut the cortical bone without penetrating), III (the screw penetrating the cortical bone). Moreover, in the present study, we also accessed the time of screws designing, the implantation time of guide wires, the insertion time of screws, the volume of blood loss, and the time of the radiation exposure (Table 2).

**Table 2:**
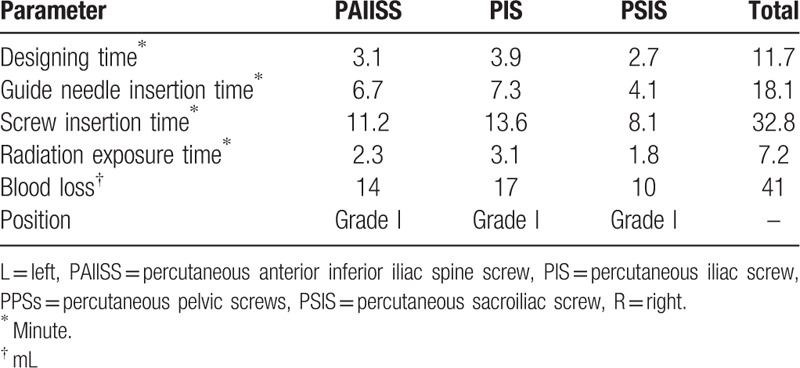
Basic characteristics of PPSs.

Postoperatively radiographs (Fig. 1B–F) and computed tomography (Fig. 2B–D) were preformed to check the position of inserted screws. The results of imaging examination show that all the 3 placed screws were classified as grade I. Besides, totally 3 percutaneous screws were inserted, the designing time of all screws was 11.7 minutes, the time of all guide needles insertion was 18.1 minutes, the time of all screws insertion was 32.8 minutes, blood loss was 21 mL, and the time of radiation exposure lasted 7.2 minutes. No surgical complications, including neurovascular compromise, wound infection, fracture nonunion, and screw loosening, were observed during the 12 months follow up visit.

## 4 Discussion

Lateral compression fractures are one of the most frequently PRI, and are usually vertically stable.^[22]^ Many factors make for the stability of these fractures even in patients with fracture to the posterior sacroiliac ligamentous complex, including the impaction of the anterior sacrum, lack of injury to the sacrotuberous ligaments, and integrity of the pelvic floor.^[23]^ This stable fracture provides the good condition for the application of navigation, because unstable fracture will lead to a serious decline in the accuracy of computer navigation. PPSs fixation is a safety and widely applied technique for the management of PRI.[^24^,^25^,^26^,^27^,^28^,^29^] However, it is technique demanding due to the complexity of the pelvis anatomy. Thus, to improve the accuracy of PPSs insertion and prevent surgical complications, we used a SVMN technique to guide screw placement.

In the present study, 3 PPSs were inserted during operation, including a PAIISS, PIS, and PSIS. Our guide needle insertion time was shorter when compared with the conclusions of previous studies.[^18^,^19^] Nevertheless, no significant difference was observed concerning the accuracy of screw placement and blood loss. Moreover, no screw loosening was found during the follow up period in this case. According to our experience, it is necessary to design the position and direction of the screw preoperatively and to use the SVMN technique intraoperatively. With regard to fluoroscopic exposure time, as previous literature reported that navigation guided Kirschner needle has a significantly shorter fluoroscopic time when compared with fluoroscopic guidance.^[30]^ In this case, PPSs were inserted successfully at the first attempt which avoids postoperative screw loosening caused by multiple insertion attempts.

Gras et al^[19]^ reported that even under the guidance of 2D navigation, PPSs insertion for PRI still has 6% incidence of screw misplacement. Consequently, precise screw insertion requires a thorough understanding of the pelvis anatomy and a good intraoperative radiograph.[^31^,^32^] In the present study, the positions of inserted screws were excellent without cortical perforation. We attribute this favorable outcome to the use of the SVMN technique for surgical designing and the screw inserting.

According to previous literatures,[^33^,^34^,^35^,^36^,^37^,^38^] the biggest advantage of SVMN technique is to improve the accuracy of percutaneous screw placement and reduce the difficulty of surgery, especially for young surgeons. Under the guidance of SVMN, surgeon adjusts the direction of the guide needle according to the screw trajectory designed preoperative. When the direction of the guide needle displayed on the display is completely consistent with the design before operation, the guide needle can be placed.[^33^,^34^,^36^,^38^] However, surgeons need to be very careful in the application of SVMN technique intraoperatively, in order to avoid the surgical complications due to inaccurate navigation. In addition, SVMN technique is only suitable for stable pelvic fractures, because unstable fracture may lead to inaccurate navigation during operation. Consequently, to prevent surgical complications, it is necessary for surgeons to master the application method of computer navigation, to familiar with the anatomy of pelvis and to select suitable patients.

In the present patient, satisfied clinical outcomes were achieved postoperatively. However, there are still limitations. The indications of SVMN technique are limited, only selected patients with stable or minimal dislocation of PRI were suitable. Moreover, SVMN has a steep learning curve, and surgeons need to spend some time studying the application of SVMN.

SVMN technique guidance PPSs insertion is an effective and safety approach for the treatment of PRI in selected patients. Besides, it is necessary for surgeons to master the rationale of computer navigation, to familiar with the anatomy of pelvis, and to select suitable patients.

## Acknowledgments

The authors are very grateful to the radiologists for their cooperation in preoperative image acquisition.

## Author contributions

**Conceptualization:** Tong Yu.

**Data curation:** Tong Yu.

**Formal analysis:** Tong Yu.

**Methodology:** Ge Huang, Tong Yu.

**Project administration:** Shuang Zheng.

**Software:** Ge Huang, Shuang Zheng.

**Supervision:** Jian-Wu Zhao.

**Validation:** Jian-Wu Zhao.

**Writing – original draft:** Bao-Ming Yuan, Shuang Zheng, Tong Yu.

**Writing – review & editing:** Tong Yu, Jian-Wu Zhao.

## References

[1] Chui KH Chan CCD Ip KC Three-dimensional navigation-guided percutaneous screw fixation for nondisplaced and displaced pelvi-acetabular fractures in a major trauma centre. Int Orthop 2018;42:1387–95. 29063184 10.1007/s00264-017-3659-z

[2] Quercetti N 3rd Horne B DiPaolo Z Gun barrel view of the anterior pelvic ring for percutaneous anterior column or superior pubic ramus screw placement. Eur J Orthop Surg Traumatol 2017;27:695–704. 27718011 10.1007/s00590-016-1864-x

[3] Dahill M McArthur J Roberts GL The use of an anterior pelvic internal fixator to treat disruptions of the anterior pelvic ring: a report of technique, indications and complications. Bone Joint J 2017;99-B:1232–6. 28860405 10.1302/0301-620X.99B9.BJJ-2016-1025.R2

[4] Gansslen A Pohlemann T Paul C Epidemiology of pelvic ring injuries. Injury 1996;27: suppl: S-A13–20. 8762338

[5] Tile M Pennal GF Pelvic disruption: principles of management. Clin Orthop Relat Res 1980;56–64. 7418324

[6] Tile M Acute pelvic fractures: I. Causation and classification. J Am Acad Orthop Surg 1996;4:143–51. 10795049 10.5435/00124635-199605000-00004

[7] Pohlemann T Bosch U Gansslen A The Hannover experience in management of pelvic fractures. Clin Orthop Relat Res 1994;69–80. 8050249

[8] Tile M The management of unstable injuries of the pelvic ring. J Bone Joint Surg Br 1995;81:941–3. 10.1302/0301-620x.81b6.10474 10615962

[9] Yang AP Iannacone WM External fixation for pelvic ring disruptions. Orthop Clin North Am 1997;28:331–44. 9208827 10.1016/s0030-5898(05)70292-3

[10] Bellabarba C Ricci WM Bolhofner BR Distraction external fixation in lateral compression pelvic fractures. J Orthop Trauma 2000;14:475–82. 11083609 10.1097/00005131-200009000-00003

[11] Dolati B Larndorfer R Krappinger D Stabilization of the posterior pelvic ring with a slide-insertion plate. Oper Orthop Traumatol 2007;19:16–31. 17345025 10.1007/s00064-007-1193-7

[12] Muller FJ Stosiek W Zellner M The anterior subcutaneous internal fixator (ASIF) for unstable pelvic ring fractures: clinical and radiological mid-term results. Int Orthop 2013;37:2239–45. 23995332 10.1007/s00264-013-2032-0 PMC3824913

[13] Gansslen A Hildebrand F Kretek C Supraacetabular external fixation for pain control in geriatric type B pelvic injuries. Acta Chir Orthop Traumatol Cech 2013;80:101–5. 23562252

[14] Winkelhagen J van den Bekerom MP Bolhuis HW Preliminary results of cannulated screw fixation for isolated pubic ramus fractures. Strategies Trauma Limb Reconstr 2012;7:87–91. 22547056 10.1007/s11751-012-0134-7 PMC3535133

[15] Peng KT Huang KC Chen MC Percutaneous placement of iliosacral screws for unstable pelvic ring injuries: comparison between one and two C-arm fluoroscopic techniques. J Trauma 2006;60:602–8. 16531861 10.1097/01.ta.0000200860.01931.9a

[16] Starr AJ Walter JC Harris RW Percutaneous screw fixation of fractures of the iliac wing and fracture-dislocations of the sacro-iliac joint (OTA Types 61-B2.2 and 61-B2.3, or Young-Burgess “lateral compression type II” pelvic fractures). J Orthop Trauma 2002;16:116–23. 11818807 10.1097/00005131-200202000-00008

[17] Thakkar SC Thakkar RS Sirisreetreerux N 2D versus 3D fluoroscopy-based navigation in posterior pelvic fixation: review of the literature on current technology. Int J Comput Assist Radiol Surg 2016;12:69–76. 27503119 10.1007/s11548-016-1465-5

[18] Wang JQ Wang Y Feng Y Percutaneous sacroiliac screw placement: a prospective randomized comparison of robot-assisted navigation procedures with a conventional technique. Chin Med J (Engl) 2017;130:2527–34. 29067950 10.4103/0366-6999.217080 PMC5678249

[19] Gras F Marintschev I Wilharm A 2D-fluoroscopic navigated percutaneous screw fixation of pelvic ring injuries--a case series. BMC Musculoskelet Disord 2010;11:153. 20609243 10.1186/1471-2474-11-153 PMC2916892

[20] Thakkar SC Thakkar RS Sirisreetreerux N 2D versus 3D fluoroscopy-based navigation in posterior pelvic fixation: review of the literature on current technology. Int J Comput Assist Radiol Surg 2017;12:69–76. 27503119 10.1007/s11548-016-1465-5

[21] Yu T Zheng S Zhang X A novel computer navigation method for accurate percutaneous sacroiliac screw implantation: a technical note and literature review. Medicine (Baltimore) 2019;98:e14548. 30762801 10.1097/MD.0000000000014548 PMC6408062

[22] Tile M Pelvic disruption: principles of management. Clin Orthop Relat Res 1980;151:56–64. 7418324

[23] Mostafavi HR Tornetta Iii P Radiologic evaluation of the pelvis. Clin Orthop Relat Res 1996;6–14. 8769431 10.1097/00003086-199608000-00003

[24] Yano S Aoki Y Watanabe A Less invasive lumbopelvic fixation technique using a percutaneous pedicle screw system for unstable pelvic ring fracture in a patient with severe multiple traumas. J Neurosurg Spine 2017;26:203–7. 27716018 10.3171/2016.7.SPINE16323

[25] Elzohairy MM Salama AM Open reduction internal fixation versus percutaneous iliosacral screw fixation for unstable posterior pelvic ring disruptions. Orthop Traumatol Surg Res 2017;103:223–7. 28017873 10.1016/j.otsr.2016.12.002

[26] Dilogo IH Fiolin J Surgical technique of percutaneous iliosacral screw fixation in S3 level in unstable pelvic fracture with closed degloving injury and morrell lavallee lesion: two case reports. Int J Surg Case Rep 2017;38:43–9. 28735116 10.1016/j.ijscr.2017.07.008 PMC5522959

[27] Bousbaa H Ouahidi M Louaste J Percutaneous iliosacral screw fixation in unstable pelvic fractures. Pan Afr Med J 2017;27:244. 28979645 10.11604/pamj.2017.27.244.11506 PMC5622821

[28] Yuan Y Wang T Yuan J Treatment of Day type pelvic crescent fracture by using percutaneous cannulated screw fixation technique. Zhongguo xiu fu chong jian wai ke za zhi = Zhongguo xiufu chongjian waike zazhi = Chin J Reparat Reconstruct Surg 2018;32:139–44. 10.7507/1002-1892.2201709002 PMC8414102 29806401

[29] Wang H Wang F Leong AP Precision insertion of percutaneous sacroiliac screws using a novel augmented reality-based navigation system: a pilot study. Int Orthop 2016;40:1941–7. 26572882 10.1007/s00264-015-3028-8

[30] Ohtori S Inoue G Orita S Comparison of teriparatide and bisphosphonate treatment to reduce pedicle screw loosening after lumbar spinal fusion surgery in postmenopausal women with osteoporosis from a bone quality perspective. Spine (Phila Pa 1976) 2013;38:E487–92. 23354115 10.1097/BRS.0b013e31828826dd

[31] Akagi M Ikeda N Fukiage K A modification of the retrograde medullary screw for the treatment of bilateral pubic ramus nonunions: a case report. J Orthop Trauma 2002;16:431–3. 12142834 10.1097/00005131-200207000-00012

[32] Altman GT Altman DT Routt ML Jr Symptomatic hypertrophic pubic ramus nonunion treated with a retrograde medullary screw. J Orthop Trauma 2000;14:582–5. 11149506 10.1097/00005131-200011000-00012

[33] Yu T Qu Y Zhang XW A screw-view model of navigation aid retrograde transpubic screw fixation for anterior pelvic ring fracture: a case report with 28 months follow-up and technical note. Medicine (Baltimore) 2018;97:e13646. 30572479 10.1097/MD.0000000000013646 PMC6320056

[34] Yu T Yang L Zheng S Screw view model of navigation in posterior corrective surgery for adolescent idiopathic scoliosis: a case report and technique note. Medicine (Baltimore) 2019;98:e14804. 30896624 10.1097/MD.0000000000014804 PMC6709026

[35] Yu T Yuan BM Zhang XW Technology of percutaneous cannulated screw implantation using screw view model of navigation in Garden type I of femoral neck fracture: a case report. Medicine (Baltimore) 2019;98:e15591. 31124935 10.1097/MD.0000000000015591 PMC6571405

[36] Zhao J Yang L Zheng S A novel screw view model of 3D navigation for upper cervical pedicle screw placement: a case report. Medicine (Baltimore) 2019;98:e15291. 31083161 10.1097/MD.0000000000015291 PMC6531100

[37] Zhao J Zhao X Yang L Percutaneous vertebroplasty with granulated allogeneic bone grafting using screw-view model of navigation for thoracolumbar compressive fracture: a case report. Medicine (Baltimore) 2019;98:e15715. 31096524 10.1097/MD.0000000000015715 PMC6531076

[38] Zhao JW Yu T Chu GY Accuracy and safety of percutaneous periacetabular screw insertion using screw view model of navigation in acetabular fracture: a case report. Medicine (Baltimore) 2018;97:e13316. 30544391 10.1097/MD.0000000000013316 PMC6310583

